# Traffic tracers in a suburban location in northern Spain: relationship between carbonaceous fraction and metals

**DOI:** 10.1007/s11356-015-5955-8

**Published:** 2016-01-22

**Authors:** L. Megido, L. Negral, L. Castrillón, E. Marañón, Y. Fernández-Nava, B. Suárez-Peña

**Affiliations:** Department of Chemical and Environmental Engineering, University Institute of Industrial Technology of Asturias, University of Oviedo, Gijón Campus, 33203 Gijón, Spain; Department of Materials Science and Metallurgiscal Engineering, Polytechnic School of Engineering, University of Oviedo, Gijón Campus, 33203 Gijón, Spain

**Keywords:** Air-mass origin, Brake wear, EUSAAR2, Non-exhaust emissions, Particulate matter, Road traffic, Trace metals

## Abstract

PM10 and black smoke were monitored at a suburban sampling station located in the northern Spanish city of Gijón. Thirty-two metals and total carbon (TC) (i.e., organic carbon (OC) and elemental carbon (EC)) were analyzed over a year. The study of air-mass origin based on 5-day back trajectories was carried out to assess its influence on the recovery data. Different strategies were implemented to infer the influence of traffic in the area. On average, TC accounted for 29 % of the PM10 fraction, with OC forming 77 % of this TC. The influence of traffic was clearly reduced during intense Atlantic advection episodes, when OC and EC decreased up to 0.39 and 0.22 μg C/m^3^, respectively. In contrast, the highest values were reported during regional episodes, exceeding 10 μg C/m^3^ of OC and 2 μg C/m^3^ of EC. The correlation between EC and OC was found to notably improve when considering the days with high traffic flow (from *R*^2^ = 0.46 to *R*^2^ = 0.74). This pattern was also reproduced by black smoke and EC (from *R*^2^ = 0.49 to *R*^2^ = 0.59). Cu and Sn were found to be reliable traffic tracers given their high dependence on EC (*R*^2^ = 0.82 and *R*^2^ = 0.79, respectively). Nevertheless, Sn, Ba, and Sb showed a better correlation with Cu than EC, suggesting a common origin. In the case of Sn, *R*^2^ improved from 0.79 to 0.91. The Cu/Sb ratio had a mean value of 6.6 which agrees with diagnostic criterions for brake wear particles. The relationships and ratios between EC, Cu, Sb, Sn, Ba, and Bi pointed out to non-exhaust emissions, playing a significant role in the chemical composition of PM10. Brake wear was presented as the most likely origin for Cu, Sb, and Sn.

**Electronic supplementary material:**

The online version of this article (doi:10.1007/s11356-015-5955-8) contains supplementary material, which is available to authorized users.

## Introduction

Different sources contribute to airborne particulate matter (PM). Certain chemical species have been scientifically proposed as tracers for road traffic, the presence of which in PM may differ depending on the type of vehicle engine and road surface abrasion (Sternbeck et al. 2002; Amato et al. 2009).

PM emissions involve carbon in different chemical and physical forms. Carbonaceous particles may account for the largest fraction of these emissions. Total carbon (TC) comprises of organic carbon (OC) and elemental carbon (EC). The latter is also called black carbon (BC), depending on the method employed in its determination. Typically, BC is determined by optical methods and EC using thermal analysis (Gelencsér 2004). OC comprises a major percentage of PM10. In contrast, the contribution of EC is somewhat lower (Negral et al. 2008). EC is a suitable indicator to assess the impact of traffic on PM (Minguillón et al. 2014). The origin of EC is exclusively primary, whereas OC may also have secondary origins (Salvador et al. 2007; Sánchez de la Campa et al. 2009; Minguillón et al. 2014). Furthermore, according to ISO 1993, black smoke (BS) is strongly light-absorbing particulate material mainly constituted by “soot,” i.e., particulates that contain carbon in its elemental form. Both BS and EC reflect the primary contribution of incomplete combustion emissions to PM (Gelencsér 2004). EC has been specifically defined as a marker for diesel exhaust (Sharma et al. 2014); therefore, abatement strategies may be focused on controlling diesel traffic emissions (Harrison and Yin 2008). Some studies showed EC as an appropriate indicator for traffic measures on air quality suggesting traffic volume, composition, and congestion as factors to control its emissions (Keuken et al. 2012).

Non-exhaust emissions (i.e., PM from brake wear, tire wear, road abrasion, and road dust resuspension) are raised in relevance due to the forthcoming reduction of exhaust pollutants (Thorpe and Harrison 2008; Fuzzi et al. 2015). Some metals have been related to non-exhaust emissions originating from road traffic using different approaches. Traffic tracers can be identified by comparing the levels of chemical elements at traffic sites and/or using source apportionment analysis. Querol et al. (2007) identified Cu–Sb as tracers of brake abrasion as their levels were relatively high in urban areas of Spain. Minguillón et al. (2014) studied trace and major elements in PM10 in different areas in Barcelona, finding higher concentrations of Ba, Cr, Cu, Fe, Mo, Zn, Mn, Pb, Sn, and Zr at traffic sites rather than in rural and urban background locations. Hueglin et al. (2005) found increasing values of several metals from urban kerbside to urban backgrounds, near-city and rural sites, inferring traffic as the main source of them.

Another approach involves finding relationships between elements in order to prove a common origin in road traffic. Pérez et al. (2010) correlated mean daily BC levels with TC, Ni, Cu, Fe, Sn, and Sb to demonstrate their related source. Vehicle emissions depend on the location. As an example, brakes contain high metal concentrations that considerably vary between brands and countries (Sternbeck et al. 2002). According to the literature, the major source of Cu is brake wear. Hence, different ratios and correlations with this metal have been carried out in a number of studies to prove a common origin: Fe/Cu, Ba/Cu, Sb/Cu, Sn/Cu, and Zn/Cu (Sternbeck et al. 2002; Querol et al. 2007; Amato et al. 2009; Pérez et al. 2010; Alves et al. 2015). These ratios vary depending on the sampling site. Grigoratos and Martini (2015) posed concerns regarding the potential adverse health effects of brake wear particles since they lie into diameters smaller than 100 nm.

TC has been associated with vehicle exhaust emissions and metals with re-suspension of road dust originating from mechanical wear and degradation of tires, brakes, and pavement abrasion. The metals emitted by vehicles have generally been determined by direct measurements and dynamometer driving cycles simulating different backgrounds (Keuken et al. 2012). As conventional dynamometric tests do not reflect real emissions of traffic, studies have been carried out in road tunnels for a more realistic approach. Although, this strategy implies the loss of other emission sources (Pio et al. 2011; Alves et al. 2015).

In this study, the PM10 fraction and its chemical composition were monitored at a suburban area located in the northern Spanish city of Gijón. BS was also measured at this location throughout the same period. The aim was to find relationships between frequently described traffic tracers in a complex scenario with multiple sources. Special attention was paid to TC and several of the previously mentioned metals. Moreover, traffic was taken into account in order to determine whether its influence was reflected in the correlations between tracers and hence discriminate the origin of the collected particulate matter. In addition, air-mass origins were studied to associate them with the related pollutant load. The variety of strategies implemented to revise the experimental data and infer the impact of traffic in the sampling station may contribute to increase the state-of-the-art in non-exhaust emissions.

## Materials and methods

### Study area

The study area was located at the University Campus in Gijón (Spain), specifically at a suburban sampling station affected by irregular road-traffic intensity. Gijón is a coastal city in the shoreline of the Cantabrian Sea. It has an Atlantic climate characterized by moderate mean temperatures, abundant rainfall from winter to early spring, and seasonal winds. The Spanish National Meteorological Agency (AEMET) has recorded main meteorological parameters in the sampling area from October 2013. Before that month, another meteorological station located within a distance of 1.7 km from the air monitoring site supplied these parameters. According to the data provided by these stations, the temperature fluctuated between −2.2 and 30.0 °C, with a minimum mean value of 10.2 °C and a maximum mean value of 19.0 °C, during the sampling period. The accumulated rainfall reached 977 mm. Winds were detected up to 13 m/s. The hours of sunshine in 2013 and 2014 were 1756 and 1833, respectively.

The municipal area of Gijón had a population density of 1518 inhabitants/km^2^ in 2014 (IDEPA 2014), although the urban zone (about 7.6 % of the total municipal area) represents 90 % of the population. The daily limit value of PM10 (50 μg/m^3^) has been exceeded at one of the local monitoring stations over the last few years. The sampling station received the influence of traffic from the university campus during the academic year and from the nearby N-632 trunk road. Several points of convergence for the region’s population are connected to the city by this road. The Gijón Science and Technology Park, three educational centers, and the local hospital are the main nerve centers of the area. Other main anthropogenic pollution sources include harbor activities (19 million tons of goods handled at Port Authority quays and berths (Port of Gijón 2014)), a cement plant (clinker kiln production capacity of 1500 t/day)^1^, a coal power station (installed power of 903 MW)^1^, and a steel production factory (blast furnaces with production capacity of 4.5 million tons a year)^1^.

### Procedure

#### Gravimetric determination of PM10

PM10 samples were collected daily from 11 July 2013 to 31 July 2014 using a high-volume sampler MCV CAV-A/MSb (30 m^3^/h) for subsequent gravimetric determination. A microbalance with a resolution of 0.01 mg was used for this purpose. The sampler was placed at a height of 3 m, which is normal practice in the Spanish Air Quality Monitoring Network, and it was in accordance with the requirements set by the European Commission (2008). The 150-mm-diameter filters were made of glass microfiber (MCV GF1-150) and quartz microfiber (Pallflex-Tissue Quartz 2500QAT-UP). The latter had superior chemical purity and low content in trace organics obtained by a heat treatment during their manufacturing process. Thus, they produce less interference on chemical determinations (EN 12341:^1998^). Fifty-two samples were collected over this matrix.

#### Analysis of chemical species

PM10 samples collected on quartz microfiber filters were digested by HF, HNO_3_, and HClO_4_ for metal extraction, as in a previous study (Negral et al. 2008). Afterward, thirty-two chemical species were determined by inductively coupled plasma mass spectrometry (ICP-MS). The same procedure was followed with blank filters and reference material from the National Institute of Standards and Technology (NIST), i.e., Standard Reference Material^®^ 1648a (Urban Particulate Matter). Errors were mostly kept below 10 %.

#### Measurement of organic and elemental carbon

Thermal-optical reflectance/transmittance methods (TOR/TOT) are used for total carbon quantification. These methods are based on the volatilization and oxidation of carbon-containing particles at different temperatures and oxidation conditions. Adsorption of volatile organic species or volatilization losses may lead to overestimation or underestimation of TC (Gelencsér 2004). Moreover, some thermal evolution protocols exist (e.g., IMPROVE, NIOSH, EUSAAR2), which adds difficulty when comparing TC results with others from literature (Vodička et al. 2015).

In the present study, the EUSAAR2 thermal evolution protocol was employed to determine OC and EC (limit of quantification of 0.1 μg C/m^3^). The following methodology was performed: a defined area of the sampling filter was cut and placed in the instrument’s oven, previously purged with helium. Initially, the temperature of the oven increased up to a first maximum (650 °C). The OC was volatilized or charred in/on the filter and pyrolytic carbon (PC) was formed. Then, the oven cools and a second temperature ramp started (up to 850 °C). CO_2_ was formed by the oxidation of EC and PC. All gases released during the process were brought into a manganese dioxide furnace where organic vapors were oxidized to CO_2_. CO_2_ was mixed with H_2_ and carried with He through a heated Ni catalyst. The Ni catalyst reduced the CO_2_ to methane. Then, the latter was measured using a flame ionization detector (FID). Internal and external carbon standards were used for calibration. The burning process of organic carbon to elemental was corrected automatically by optical transmittance (TOT). Therefore, underestimation of OC and the corresponding overestimation of EC were avoided. The temperature set points and residence times of the EUSAAR2 protocol were provided by Cavalli et al. (2010). These researchers described this protocol regarding potential European standard procedures to measure the carbonaceous aerosol fraction.

#### Determination of black smoke

BS was also sampled daily throughout the same period using a low-volume captor CPV-8D/A (2 m^3^/day) and Whatman No. 1 filters (47-mm diameter). BS was determined using an EEL Model 43D Digital Smokestain Reflectometer. The percentage of reflectance produced by the sampling stain was determined by the detector and then related to BS concentration or the absorption coefficient (α) by the procedures of the Organization for Economic Cooperation and Development (OECD) method (OECD 1964) or the ISO 1993 standard (ISO 1993), respectively.

### Air-mass origins

**Fig. 1:**
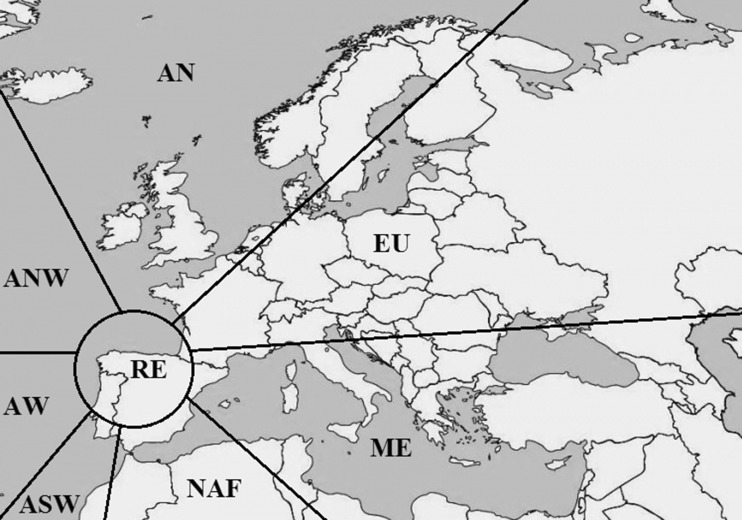
Classification of air-mass origins: Northern Atlantic (AN); European (EU); Mediterranean (ME); Northern African (NAF); South-western Atlantic (ASW); Western Atlantic (AW); North-western Atlantic (ANW); and Regional (RE)

The HYSPLIT Model developed at the NOAA Air Resources Laboratory (Draxler and Hess 1997) was run to calculate 120-h isentropic back trajectories at three different heights (750, 1500, and 2500 m above sea level) in order to catalog air-mass origins at the sampling station (43° 31′ 23″ N, 5° 37′ 16″ W) according to eight different sectors (Fig. 1): Northern Atlantic (AN), North-western Atlantic (ANW), Western Atlantic (AW), South-western Atlantic (ASW), Northern African (NAF), Mediterranean (ME), European (EU), and Regional (RE).

## Results and discussion

### PM10 levels

Throughout the entire sampling period, PM10 levels were in the range of 4.7–69.5 μg PM10/m^3^. The mean value was 19.1 ± 10.1 μg PM10/m^3^ (*n* = 375 samples), which agreed with the urban annual mean levels (9–23 μg PM10/m^3^ in 2009) recorded in locations of northern Spain (Aldabe et al. 2011). Comparing these results with others across Europe, PM10 levels at the university campus in Gijón were in the same range as those from Central Europe, where they generally varied from 19 to 24 μg PM10/m^3^ (Pey et al. 2009; Putaud et al. 2010; EEA 2014; Fuzzi et al. 2015).

**Fig. 2:**
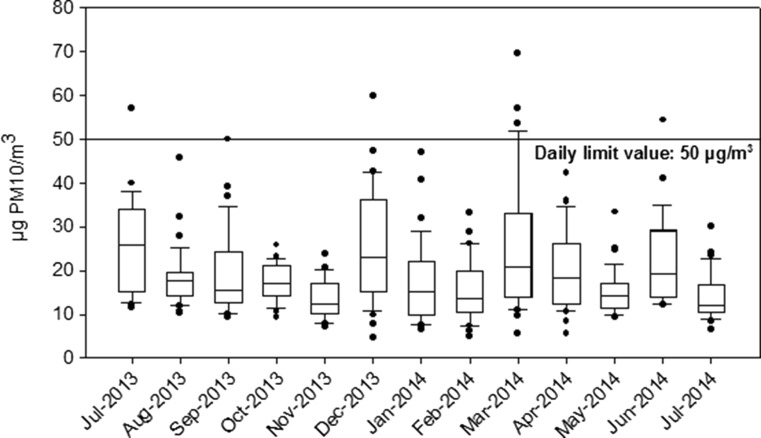
Box plot of PM10 levels showing outliers from 10th to 90th percentiles

**Fig. 3:**
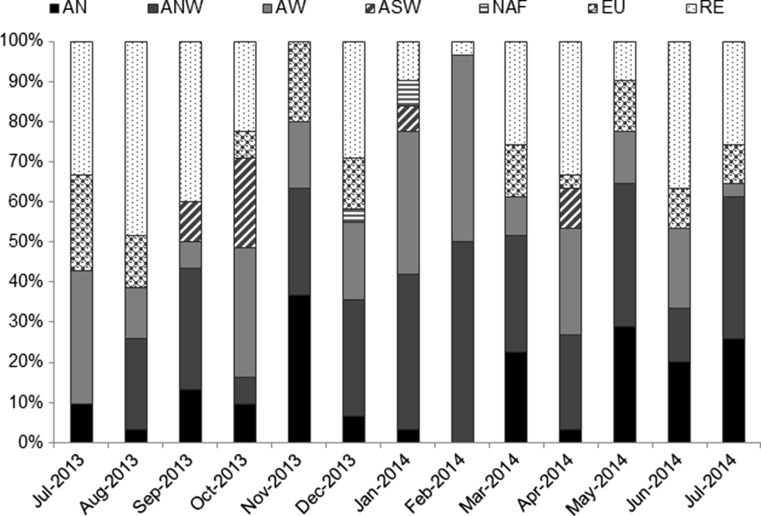
Contribution of air-mass origins over the sampling period

Figure 2 shows a box plot of the PM10 levels. The distribution of the monthly PM10 levels was not symmetrical, being the median below the average in most cases. The daily limit value was exceeded on eight occasions. On those days, the precipitation rate was negligible (below 1.0 mm). July and December 2013 and March 2014 presented the greatest dispersion of the recorded data (Fig. 2). In July 2013, air masses from AW, RE, and EU were predominant (Fig. 3), with high differences in the mean PM10 levels (AW 13.8 ± 1.8 μg PM10/m^3^; RE 34.1 ± 11.7 μg PM10/m^3^, and EU 32.0 ± 4.2 μg PM10/m^3^, respectively), which explained the dispersion of the overall data during this month. Similarly, December 2013 and March 2014 were characterized by great variation between the mean PM10 levels recorded under the predominant air-mass origins: ANW and RE (Fig. 3). In December 2013, their averages were 19.0 ± 10.5 and 37.9 ± 6.6 μg PM10/m^3^, respectively. In March 2014, the mean PM10 was 14.7 ± 6.2 μg PM10/m^3^ under ANW and 27.4 ± 7.5 μg PM10/m^3^ under RE episodes. Additionally, although EU air masses represented 13 % of the days of March 2014 (Fig. 3), the average PM10 levels with these origins (55.9 ± 10.9 μg PM10/m^3^) were well above the latter mentioned (i.e., ANW and RE). Seven exceedances of the daily limit occurred during these 3 months (Fig. 2). It should be noted that most of these levels occurred on weekdays, which points to traffic origin. Furthermore, the highest levels were under NAF (59.9 μg PM10/m^3^ on 12 December 2013) and EU (69.5 μg PM10/m^3^ on 13 March 2014 and 57.1 μg PM10/m^3^ on 14 March 2014) episodes. Therefore, although high PM10 levels were usually recorded under RE episodes, the maximum values were related to long transport processes in the absence of rainfall. Likewise, high PM10 levels were observed in winter at urban and traffic locations in northern Spain (Pamplona), which Aldabe et al. (2011) associated to low precipitation and dilution, as well as air-mass transport from Europe.

Some authors have found considerable variances in PM10 levels depending on the season (Umlauf et al. 2010; Schilirò et al. 2015). Sánchez de la Campa et al. (2009) explained the higher PM10 levels recorded in summer rather than in winter by the high photochemical activity during that period. However, the data of the present study showed no major differences. In the cold period (October–March), PM10 ranged between 4.7 and 69.5 μg PM10/m^3^, with an average of 19.3 ± 11.2 μg PM10/m^3^, whereas the warm period (April–September) varied between 5.6 and 57.0 μg PM10/m^3^ and the average was 18.8 ± 9.1 μg PM10/m^3^. This was not surprising due to the region’s Atlantic climate and low number of hours of sunshine (1756 h in 2013 and 1833 h in 2014).

### Carbonaceous particles

**Fig. 4:**
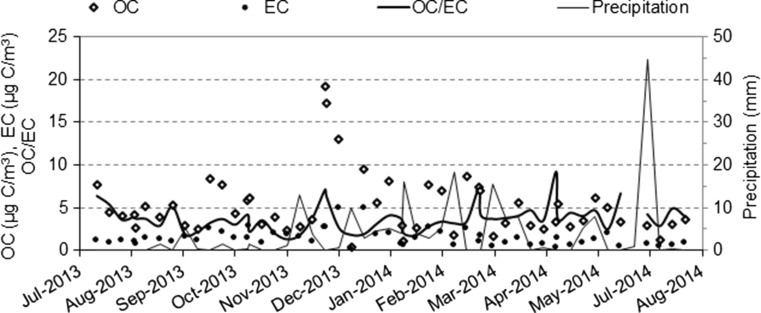
Variation in TC and precipitation between July 2013 and July 2014

Table 1 shows the seasonal variations of EC and OC and the EC/TC ratio which was similar in warm and cold periods. Greater OC levels are generally recorded in winter than in summer in most European locations, which is related to residential heating (i.e., biomass and fossil fuel combustion) and stagnant meteorological conditions (Fuzzi et al. 2015). Indeed, the maximum EC concentration (4.96 μg C/m^3^) was observed during the cold period. The variability in EC, OC, and precipitation during the sampling period is shown in Fig. 4. On average, TC accounted for 29 ± 13 % of the PM10 fraction with OC forming 77 ± 7 % of this TC. OC was hence an important contributor to PM mass, as expected from literature (Putaud et al. 2010; Bisht et al. 2015; Chow et al. 2015; Fermo et al. 2015). The increase in the contribution of TC to PM10 (from 24.8 to 33.2 %) from warm to cold period enhanced its importance as a component of PM10. Negral et al. (2008) found organic matter and elemental carbon representing 18 % of PM10 in southeastern Spain (Cartagena). These researchers discussed the relationship between the carbonaceous fraction and traffic from a highway and coach and train stations close to the sampling station.

**Table 1:** Mean, minimum, and maximum values of OC, EC, OC/EC, EC/TC in PM10 during warm and cold periods (*n* = 52) between July 2013 and July 2014

	Entire sampling period (*n* = 52)			Warm period: April–September (*n* = 26)			Cold period: October–March (*n* = 26)		
	Mean	Min.	Max.	Mean	Min.	Max.	Mean	Min.	Max.
OC (μg C/m^3^)	5.12 ± 3.62	0.39	19.23	4.06 ± 1.65	1.27	8.44	6.18 ± 4.66	0.39	19.23
EC (μg C/m^3^)	1.45 ± 0.99	0.22	4.96	1.05 ± 0.51	0.36	2.61	1.85 ± 1.19	0.22	4.96
OC/EC	3.79 ± 1.58	1.30	9.22	4.21 ± 1.52	1.90	9.22	3.38 ± 1.55	1.30	7.32
EC/TC	0.23 ± 0.07	0.10	0.43	0.21 ± 0.05	0.10	0.35	0.25 ± 0.08	0.12	0.43

During the warm period, RE and ANW episodes were predominant (Fig. 3), constituting the most frequent air-mass origin in this period (32.4 and 24.0 %, respectively). In the cold period, however, ANW and AW were the most frequent episodes (29.7 and 26.4 %, respectively), highlighting the importance of Atlantic advections. The influence of traffic at the station clearly diminished during these episodes, when OC decreased up to 0.39 μg C/m^3^ and EC up to 0.22 μg C/m^3^. These values, which occurred on 25 December 2013, might be explained by low traffic flow in the area. Vehicular displacements from the city and its environs to the working and educational centers close to the sampling station were reduced as that day was a national public holiday. According to a nearby traffic measuring point located on the N-632 trunk road, the average daily traffic (ADT) from Monday to Friday was 10,329 vehicles/day in 2013. On Wednesday, 25 December 2013, the ADT was reduced to 4416 vehicles/day.

In contrast, the highest TC values were generally reported during RE episodes, exceeding 10 μg C/m^3^ for OC and 2 μg C/m^3^ for EC. On these days, PM10 levels were also considerably above the average (19.1 μg PM10/m^3^), up to 42.6 μg PM10/m^3^. Higher EC, OC, and PM10 values were expected during RE episodes due to poor air circulation (Negral et al. 2012). Moreover, no precipitation was recorded on these days.

The OC/EC ratio varies daily from 1.30–9.22 (Table 1), with an average of 3.79 ± 1.58. This mean value was similar to the results reported by other authors in urban locations or sites influenced by road traffic in Spain (OC/EC = 2.3), Helsinki (OC/EC = 3.23), Coimbra (OC/EC = 2.75), and Delhi (OC/EC = 4.38) (Harrison et al. 1997; Viidanoja et al. 2002; Sánchez de la Campa et al. 2009; Sharma et al. 2014). Road traffic was the most relevant source for the chemical composition of the airborne PM in all those sites. Besides, the lowest OC/EC ratios were registered during the cold period, which is frequent in urban areas during days with low photochemical activity when the contributions of secondary OC are less important to the OC aerosol (Pio et al. 2011).

**Fig. 5:**
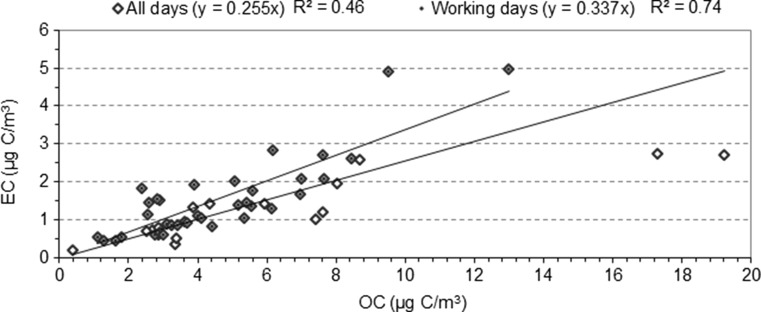
Comparison of the correlation between EC and OC considering all sampling days (*n* = 52) and only working days (*n* = 34)

EC and OC concentrations presented a moderate correlation, i.e., *R*^2^ = 0.46 (Fig. 5), which improved slightly when it did not pass through the origin (*R*^2^ = 0.53). The relevance of traffic at the sampling station was demonstrated when days with high traffic flow (i.e., working days) were independently studied. Working days (*n* = 34) were taken into account to discriminate these periods as traffic was markedly lower on weekends and public holidays than on weekdays. In 2013, the ADT was 10,329 vehicles/day from Monday to Friday, 7148 vehicles/day on Saturdays, and 6871 vehicles/day on Sundays. Similar data was registered in 2014 (ADT of 10,425 vehicles/day from Monday to Friday, 7214 vehicles/day on Saturdays, and 6947 vehicles/day on Sundays). An improvement in the results of this study was shown by the linear relationship between EC and OC (*R*^*2*^ = 0.74) (Fig. 5), indicating that road traffic was a major common source. Keuken et al. (2012) also found a different composition in PM10 on Sundays due to reduced traffic flow. Sharma et al. (2014) reported the influence of vehicular emissions on PM10 with a linear trend between OC and EC (*R*^2^ = 0.53).

The influence of traffic on pollution levels was also reproduced via the correlation between BS and EC, although to a lesser extent than the correlation between EC and OC, i.e., from *R*^2^ = 0.49 (all days) to *R*^2^ = 0.59 (working days). BS concentration was calculated on the basis of measured values of reflectance in agreement with the OECD standard method. The OECD (1964) relates BS with the reflectance of a stain in a filter. Heal and Quincey (2012) reported that the ISO (1993) standard provides an expression to calculate α from reflectance values, which is compatible with the early OECD standard method after applying a scaling factor. The α-EC correlation thus provided similar results to the BS-EC correlation, as expected (*R*^2^ = 0.40), although the effect of excluding the days with low traffic flow was more notable (*R*^2^ = 0.57).

### Elemental carbon and metals

**Fig. 6:**
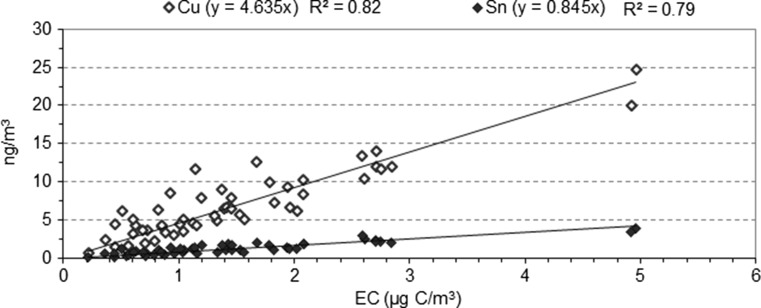
Correlations of Cu and Sn with EC

As stated in the “Introduction” section, the correlation between some metals and EC may indicate that road traffic is a common source. Relevant results were found in Cu (Fig. 6), in which a linear regression showed its high dependence on EC (*R*^2^ = 0.82). Sn similarly reproduced this pattern with a good fit (*R*^2^ = 0.79) (Fig. 6). All data were considered in these relationships, without distinguishing working days. Along these same lines, Pérez et al. (2010) pointed to traffic-related sources for these elements by correlating them with BC (*R*^2^ = 0.58 for Cu and *R*^2^ = 0.66 for Sn).

**Fig. 7:**
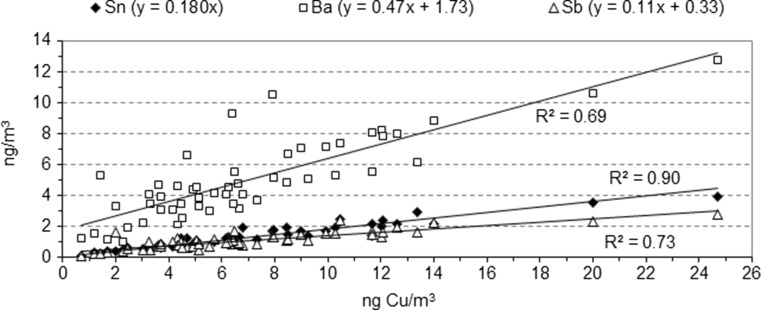
Correlations of Sn, Ba, and Sb with Cu

Some traffic tracers presented a better correlation with Cu than with EC, suggesting a common origin with the former. This was the case of Sn, whose correlation improved from *R*^2^ = 0.79 with EC (Fig. 6) up to *R*^2^ = 0.91 with Cu (Fig. 7), as well as Ba (EC–Ba: *R*^2^ = 0.53; Cu–Ba: *R*^2^ = 0.69) and Sb (EC–Sb: *R*^2^ = 0.60; Cu–Sb: *R*^2^ = 0.73) (Fig. 7).

Thorpe and Harrison (2008) reviewed studies of non-exhaust PM from traffic, revealing Cu, Sb, Ba, and Sn as compounds of brakes. Indeed, Cu and Sb were presented as reliable tracers of brake wear. The Cu/Sb ratio had a mean value of 6.6 (Table 2). Typical reported values of the Cu/Sb ratio range between 4.9 and 8 (Alves et al. 2015). Amato et al. (2009) studied PM10 in road dust deposited at an urban background. These authors showed a high correlation between Cu and Sb and proposed a diagnostic criterion for brake wear particles: Cu/Sb = 7.0 ± 1.9. Sternbeck et al. (2002) determined a Cu/Sb ratio of 4.6 ± 2.3 in tunnels. Nevertheless, the conclusion in both studies was that brake linings were a common source of Cu and Sb. The Cu/Sb ratio of the present study agrees with the criteria established by previous authors. Therefore, brake wear seems to have been a relevant origin for Cu and Sb in the present study. According to Grigoratos and Martini (2015), brake linings contain 1–5 % Sb in the form of stibnite (Sb_2_S_3_), which is employed as a lubricant in order to reduce vibrations and improve friction stability. Other possible origins of Sb are crust and metallurgical processes, although the diagnostic Cu/Sb ratios for these origins (125 and 10, respectively) are markedly different from the Cu/Sb ratio of brake wear (Thorpe and Harrison 2008).

**Table 2:** Relevant ratios and linear correlation coefficients (*R* ^2^) between metals as traffic tracers in PM10 (*n* = 52)

Metals	*R* ^2^	Ratio		
		Mean	Min.	Max.
Cu–Sb	0.73	6.55 ± 2.00	1.26	13.12
Cu–Sn	0.91	5.38 ± 1.02	3.60	8.39
Cu–Ba	0.69	1.40 ± 0.47	0.27	2.29
Cu–Bi	0.69	61.93 ± 21.45	13.86	106.17
Ba–Cu	0.69	0.85 ± 0.51	0.44	3.68
Ba–Sb	0.61	5.41 ± 3.88	2.07	22.97
Ba–Bi	0.69	48.50 ± 23.35	17.68	156.86
Ba–Sn	0.66	4.58 ± 3.03	2.07	22.00

Cu, Sb, and Bi were also related to brake abrasion (Minguillón et al. 2012). In fact, a relationship between Cu and Bi was found (Table 2). The high ratio between the two was due to the low values of Bi. Ba also correlated with the aforementioned elements (Table 2) and has been associated to brake wear (Garg et al. 2000). Moreover, the Ba/Cu ratio (0.85) is close to the mean value determined in tunnels, e.g., Ba/Cu = 0.75 (Sternbeck et al. 2002; Alves et al. 2015). However, the presence of Bi and Ba in PM has also been associated with metallurgical activities and mineral matter from soil, respectively (Querol et al. 2007; Rogula-Kozłowska et al. 2015). Therefore, other relevant origins of Bi and Ba cannot be discarded with the current information.

The aforementioned correlations between these chemical species (i.e., Cu, Sn, Sb, Ba, and Bi) stood at a similar degree independently of the traffic flow (e.g., Cu–Sn in working days: *R*^2^ = 0.90 (*n* = 35) versus in non-working days: *R*^2^ = 0.93 (*n* = 17)). Thus, a main common origin was gathered. However, the maximum and minimum concentrations of Cu, Sn, Sb, Ba, and Bi were determined in working and non-working days, respectively. Indeed, their average concentrations were slightly higher during working days, especially in the case of copper (i.e., 7.6 ± 4.9 μg Cu/m^3^ in working days versus 5.6 ± 3.7 μg Cu/m^3^ in non-working days), pointing to traffic as their relevant source.

Additional data are given in Online Resource 1.

## Conclusions

PM10 levels and the chemical composition of this fraction were determined at a suburban sampling station in the north of Spain. No major seasonal variation was found in PM10 at the site between cold (October–March) and warm (April–September) periods. Different strategies were implemented to study the influence of traffic in a complex scenario where other anthropogenic sources (i.e., industrial activities) played an important role in air pollution.

The OC/EC ratio was similar to those reported in the literature at urban locations or sites influenced by road traffic. Noteworthy results were found in the correlation between OC and EC (*R*^2^ = 0.46), which reflected a considerable improvement when considering exclusively days with high traffic flow (*R*^2^ = 0.74). BS similarly reproduced this pattern, confirming the relevance of carbonaceous particles in PM10. The study of the linear relationships between several metals typically accepted as traffic tracers also led to conclusions in this respect. Cu and Sn were mainly emitted by traffic given their high dependence on EC (*R*^2^ = 0.82 and *R*^2^ = 0.79, respectively). Moreover, Sn, Sb, Ba, and Bi presented a stronger correlation with Cu than with EC, pointing to a common origin for these metals. The Cu/Sb ratio was in full agreement with results from other studies, and brake wear may constitute their main source. Bi and Ba also seemed to have the same origin and were related to EC and Cu, though to a lesser extent.

As inferred from the relationships and ratios between traffic tracers, non-exhaust emissions could have played a relevant role in PM10. Brake wear was presented as the most likely origin for Cu, Sb, and Sn.

## Electronic supplementary material

Below is the link to the electronic supplementary material.

ESM 1 (PDF 21 kb)

## Footnotes

1 Information available at The European Pollutant Release and Transfer Register (E-PRTR)
