# Supplementary material for: Traffic tracers in a suburban location in northern Spain: relationship between carbonaceous fraction and metals
Source: Environ Sci Pollut Res Int. 2016 Jan 22;23:8669–78. doi: 10.1007/s11356-015-5955-8 (PMC4850174; doi:10.1007/s11356-015-5955-8)
Supplement: Supplementary file 1 — (PDF 21 kb) [file 11356_2015_5955_MOESM1_ESM.pdf]

Traffic tracers in a suburban location in northern Spain: relationship between carbonaceous fraction and metals

L. Megido[1], L. Negral[1*], L. Castrillón[1], E. Marañón[1], Y. Fernández-Nava[1], B. Suárez-Peña[2]
[1]Department of Chemical and Environmental Engineering, University Institute of Industrial Technology of Asturias, Gijón Campus, University of Oviedo, 33203 Gijón, Spain
[2]Department of Materials Science and Metallurgical Engineering, Polytechnic School of Engineering, Gijón Campus, University of Oviedo, 33203 Gijón, Spain

*Corresponding author: E-mail: negralluis@uniovi.es, Tel: +34 985182385, Fax: +34 985182337

Mean, minimum and maximum values of the chemical species found in PM10 between July 2013 and July 2014 (n = 52):

Component	Concentration (ng/m^3)						
	Mean	Min.	Max.		Mean	Min.	Max.
Na	1006 ± 785	69	3724	Ni	1.5 ± 3.3	<0.01	24
Fe	467 ± 344	78	1660	Mo	1.4 ± 2.7	<0.01	11
Ca	278 ± 187	2.4	931	Sn	1.4 ± 0.9	0.1	3.9
Mg	184 ± 110	45	510	Sb	1.1 ± 0.6	0.1	2.7
K	171 ± 96	32	567	Se	0.6 ± 0.4	<0.01	1.8
Al	132 ± 100	5.6	476	Rb	0.6 ± 0.4	0.1	2.1
Zn	46 ± 38	2.8	226	As	0.5 ± 0.3	0.1	2.0
Mn	11 ± 11	0.9	61	Cd	0.3 ± 0.4	<0.01	2.8
Pb	8.0 ± 6.2	0.6	25	Ce	0.3 ± 0.2	0.01	0.8
Ti	7.6 ± 5.8	0.7	33	Li	0.2 ± 0.1	<0.01	0.4
Cu	6.8 ± 4.6	0.7	25	La	0.1 ± 0.1	0.01	0.4
Zr	5.3 ± 2.9	0.3	10	Tl	0.1 ± 0.1	0.01	0.5
Ba	5.0 ± 2.6	1.0	13	Bi	0.1 ± 0.1	0.01	0.3
Cr	4.3 ± 3.0	0.4	12	Co	0.1 ± 0.1	0.01	0.6
Sr	1.8 ± 1.0	0.01	4.1	Be	0.01 ± 0.01	<0.01	0.04
V	1.7 ± 1.2	0.1	7.5	Ta	<0.01	<0.01	0.01
